# Sexuality in Persons With Hidradenitis Suppurativa: Factors Associated With Sexual Desire and Functioning Impairment

**DOI:** 10.3389/fpsyt.2021.729104

**Published:** 2021-10-07

**Authors:** Rossella Mattea Quinto, Simona Mastroeni, Francesca Sampogna, Luca Fania, Roberta Fusari, Luca Iani, Damiano Abeni

**Affiliations:** ^1^Clinical Epidemiology Unit, Istituto Dermopatico dell'Immacolata, Dermatological Research Hospital (IDI-IRCCS), Rome, Italy; ^2^Department of Human Sciences, European University of Rome, Rome, Italy; ^3^First Dermatological Unit, Istituto Dermopatico dell'Immacolata, Dermatological Research Hospital (IDI-IRCCS), Rome, Italy

**Keywords:** dermatology, acne inversa, sexual functioning, skin-related quality of life, anxiety, psychological distress, general health

## Abstract

Hidradenitis Suppurativa (HS) is a chronic skin disease involving intimate and sensitive areas and affecting physical and mental health. We investigated the prevalence of sexual desire and functioning impairment, and their associations with quality of life, anxiety, depression, minor psychiatric disorders (MPD), and clinical features (e.g., disease severity) in 77 patients with HS who completed self-report measures and answered to questions assessing socio-demographic characteristics, lifestyle habits, and hindered sexuality due to HS. The majority of patients reported hindered sexuality, and poor sexual functioning, while showing good levels of dyadic and solitary sexual desire. No associations were found between clinical severity and sexuality measures. Multivariate analyses showed significant associations of sexual outcome measures with alcohol consumption, low Body Mass Index, family history of HS, and severe skin symptoms. Moreover, we found that the presence of negative psychological factors (i.e., MPD, anxiety, poor mental status) increased the risk of sexual impairment. These findings underline the important role of psychological and sexual aspects in HS patients and suggest that physicians should consider the effect of disease burden on patients' sexual health.

## Introduction

Hidradenitis Suppurativa (HS) is a chronic, inflammatory, progressive, debilitating skin disease, characterized by painful, deep-seated, inflamed lesions, including nodules, sinus tracts, and malodourous abscesses (1). Literature reviews of previous studies (2, 3) suggested that HS causes a significant impairment of patients' quality of life (QoL) and that depression and anxiety are common comorbidities in individuals with HS. Moreover, people with HS reported high levels of alexithymia and psychological distress (4) as well as the worst quality of life among different skin conditions (5). Finally, due to the localization of lesions in sensitive and intimate body areas, HS may cause embarrassment and shame (5, 6) that hinder sexual activities (7).

Sexuality refers to “perceptions about one's body; the need to touch and connect with others, in both intimate and social settings; interest and ability to engage in sexual behaviors; communication of one's feelings and needs to others; and the ability to engage in satisfying sexual behaviors” (8). Impairment in sexual functioning can cause different types of maladjustment, including frustration, anxiety, depression, and damaged relationships with partners (9). Most skin diseases may have negative effects on patients' self-perception, body image, and social relationships, causing impairment in sexual functioning (10, 11). The study of Sampogna et al. (12) found that about a quarter of dermatological patients reported sexual problems, with 66.7% of HS patients having the highest level of sexual impairment. This study also showed that sexual problems in the whole sample were strongly associated with depression, anxiety, and suicidal ideation, were generally more frequent in younger patients, and were positively correlated with clinical severity and itch.

Some studies (13, 14) investigated the role of HS in patients' sexual functioning, which “refers to the normal physiological and performance standards” including “sexual desire, sexual arousal, pain-free intercourse, and orgasm” (15). Recently, Cuenca-Barrales et al. (16) reviewed the scientific evidence on HS and sexual health showing that the prevalence of sexual dysfunction in HS patients ranged from 51 to 62% and that potential risk factors for sexual dysfunction were disease activity, symptoms and the absence of a stable relationship. The authors found that an increased frequency of anxiety and depression in HS patients could represent a vicious circle in which sexual functioning impairment can cause, and is consequence of, mood disorders (16).

To the best of our knowledge, little is known about HS patients' sexual desire, which is an important aspect of sexuality (15). According to a biopsychosocial approach, sexual desire can be defined as “the sum of the forces that lean us toward and push us away from sexual behavior” (17). Female Sexual Interest/Arousal Disorder is defined as “Lack of, or significantly reduced, sexual interest/arousal” (18), as manifested by at least three criteria, with a minimum duration of symptoms of 6 months that cause clinically significant distress in individuals. Instead, the Diagnostic and Statistical Manual of Mental Disorders, fifth edition (DSM-5) defines Male Hypoactive Sexual Desire Disorder as a “Persistently or recurrently deficient (or absent) sexual/erotic thoughts or fantasies and desire for sexual activity,” with the same duration and impact as females (18). Spector, Carey and Steinberg (19) described sexual desire as the thoughts that may prompt individuals to search for or be open to sexual opportunities. These authors also proposed a multidimensional conceptualization of sexual desire, encompassing two domains: interest in dyadic sexual behavior (e.g., desired frequency of intercourse) and interest in individual sexual behavior (e.g., desired frequency of masturbation). The aim of this study was to investigate the prevalence of sexual desire and functioning impairment, and their relationships with QoL, anxiety, depression, psychological distress, and clinical features in patients with HS.

## Materials and Methods

### Sample

Seventy-seven consecutive outpatients with HS, attending the Istituto Dermopatico dell'Immacolata (IDI-IRCCS, Italy) between January 2018 and February 2019, were enrolled at the dedicated HS outpatients' clinic and included in this cross-sectional study. Inclusion criteria were: (1) age 18 years or more; (2) a new diagnosis of HS, or attending the hospital for the first time; (3) history of at least 6 months of HS symptomatology; (4) written, informed consent. Exclusion criteria were: (1) presence of lesions associated with HS in absence of other criteria to fulfill the HS diagnosis; (2) presence of major diseases of the central nervous system; (3) presence of other dermatological diseases or medical comorbidities (e.g., hypertension, diabetes mellitus, or immune-mediated diseases); (4) current major psychiatric disorders; (5) denial of informed consent. The new diagnosis of HS, the history of previous symptoms, and the presence of HS lesions were made by a board-certified dermatologist. The presence of major medical diseases and psychiatric disorders was based on clinical judgment and retrospective assessments. The study was approved by the local Ethics Committee and was carried out in accordance with the 1964 Helsinki Declaration.

### Measures

All participants completed a checklist assessing socio-demographic variables, smoking status (“never,” “current,” “ex-smokers” defined as those who have quit smoking for at least 6 months), alcohol consumption (yes/no), and measured height and weight to calculate Body Mass Index (BMI). Disease severity was assessed using the Hurley staging system (20), the Sartorius score (21), and the International HS Severity Scoring System (IHS4) (22). Pain experienced during the previous week was assessed with the Visual Analog Scale (range 0-10) (23). Other clinical information, including family history, age at onset, disease duration, and number and type of locations involved, was obtained from medical records.

Different measures were used to assess sexual functioning, sexual desire, and hindered sexuality after HS diagnosis. Sexual functioning was assessed using the 19-item Sexual Dysfunction Questionnaire (SDQ). Answers are given on 5-point scale (1: always; 5: never) and a score ≥45 indicates the presence of sexual dysfunction (24). A sample item is “I am satisfied with my sex life.” Sexual desire was measured with the Sexual Desire Inventory-2 (SDI-2) (19). The questionnaire consists of 14 items, which are rated on a Likert scale, and of two subscales: dyadic sexual desire (items 1-9; maximum score 79) and solitary sexual desire (items 10-14; maximum score 44). Higher scores indicate greater sexual interest. Patients' hindered sexuality was measured using one question with dichotomous answer categories (yes/no): “Is your sexuality hindered by your Hidradenitis Suppurativa?.”

Skin-related QoL was assessed with the Skindex-17 questionnaire (25), which measures symptoms and psychosocial aspects. Answers are given on a three-point scale (0: never; 1: rarely/sometimes; 2: often/always), with higher scores indicating a worst skin-related QoL. The 12-item General Health Questionnaire (GHQ-12) was used to assess symptoms of minor psychiatric disorders. Answers are given on a four-point scale. Individuals with scores of 4 or more based on the dichotomous scoring system (0-0-1-1) are defined as GHQ-cases (26). The Hospital Anxiety and Depression Scale (HADS) was used to assess anxiety and depression symptoms (27). Both anxiety and depression subscales include seven items, each of which are rated on a four-point scale (from 0 to 3) with total scores ranging from 0 to 21. A cutoff of 8 on each subscale is used to identify borderline cases, while a cutoff of 11 is used for identifying clinically significant symptoms of both anxiety and depression. General health status was measured using the 36-Item Short Form Health Survey (SF-36) (28). It includes eight dimensions, which are combined to provide a Physical Component Summary (PCS) and a Mental Component Summary (MCS). Scores for each domain range from 0 to 100, with higher scores indicating better health status.

### Statistical Analysis

The characteristics of HS patients were described as means and standard deviations (SD), medians and Interquartile Ranges (IQR) for continuous variables, and as absolute and relative frequencies for categorical variables. Since no cutoffs have been previously determined for the SDI-2, tertiles were calculated for each of the two subscales of the instrument. The lowest tertile (T1) was used as an indicator of low dyadic sexual desire (≤ 42) and low solitary sexual desire (≤ 2), respectively.

To explore potential factors associated with sexual dysfunction, we carried out uni- and multi-variate analyses for each of the four indices: sexual functioning (presence vs. absence), dyadic and solitary sexual desire (T1 vs. T2-T3), and hindered sexuality (yes vs. no). In the multivariate analysis we used four separate logistic regression models to estimate Odds Ratio and 95% Confidence Intervals, each for a single index as the outcome. Variables significantly associated with the outcomes in the univariate analysis (*p* < 0.10; see Supplementary Tables 1a–c) were included in the multivariate analyses. Starting with sex and age, the likelihood ratio test was used for a forward variable selection in the model. Statistical analyses were performed using STATA, release 15 (StataCorp LLC, College Station, TX).

## Results

Table 1 provides descriptive statistics on socio-demographic and clinical characteristics of the 77 HS patients. Participants had a mean age of 33.4 years (SD = 10.6). The majority of HS patients were females (64.9%), were overweight or obese (59.2%), were current or ex-smokers (81.8%), did not exercise (70.1%), were classified as Hurley II e III (67.1%), and reported severe disease according to IHS4 (52.6%).

**Table 1:** Socio-demographic and clinical characteristics of 77 patients with Hidradenitis Suppurativa.

	**N^†^ (%)**
**Sex**	
Males	27 (35.1)
Females	50 (64.9)
**Age, years**	
Mean *(SD)*	33.4 (10.6)
Median (IQR)	31 (25-41)
**Education level, years**	
Low (≤ 8)	22 (28.6)
Medium (9-13)	34 (44.2)
High (>13)	21 (27.3)
**BMI (kg/m** ^**2**^ **)**
≤ 24.9	31 (40.8)
25-29.9	26 (34.2)
≥30	19 (25.0)
**Smoking status**	
Never smokers	14 (18.2)
Current smokers	54 (70.1)
Ex-smokers (at least 6 m)	9 (11.7)
**Alcohol use**	
No	49 (63.6)
Yes	28 (36.4)
**Physical activity**	
No	54 (70.1)
Yes	23 (29.9)
**Age at onset**	
<18	32 (41.6)
≥18	45 (58.4)
**Disease duration, y**	
Mean *(SD)*	11.2 (8.2)
Median (IQR)	10 (5-15)
**Family history of HS**	
No	68 (88.3)
Yes	9 (11.7)
**Hurley stage**	
I	25 (32.9)
II	45 (59.2)
III	6 (7.9)
**Sartorius score**	
0-49	44 (57.9)
50-99	20 (26.3)
≥100	12 (15.8)
**IHS4**	
Mild ( ≤ 3)	14 (18.4)
Moderate (4-10)	22 (29.0)
Severe (≥11)	40 (52.6)
**Pain (VAS)**	
Mean *(SD)*	5.3 (3.2)
**Number of locations involved**	
Mean *(SD)*	2.7 (1.5)
**Involvement of:**	
Groin	62 (81.6)
Axilla	50 (65.8)
Gluteal	32 (42.1)
Mammary region	15 (19.7)
Neck	10 (13.2)
Other^‡^	10 (13.2)

*BMI, body mass index; HS, Hidradenitis Suppurativa; SD, Standard Deviation; IQR, Interquartile Range; IHS4, International HS Severity Score System; VAS, Visual Analog Scale.*

† *Totals vary because of missing values.*

‡ *back, sacred region, face*.

Table 2 described variables related to sexual health, skin-related QoL, health status, psychological distress, anxiety and depression. The majority of HS patients reported hindered sexuality and sexual dysfunction (61.8 and 60.8%, respectively). The mean values of dyadic and solitary sexual desire measured with the SDI-2 were 43.3 (SD = 16.5) and 8.9 (SD = 8.3), respectively. Our results showed that disease severity and clinical characteristics were not significantly associated with any sexuality measures (see Supplementary Table 1b).

**Table 2:** Sexual health, quality of life, health status, psychological distress, anxiety and depression in 77 patients with Hidradenitis Suppurativa.

	**N^†^(%)**
**Hindered sexuality**	
No	29 (38.2)
Yes	47 (61.8)
**Sexual dysfunction (SDQ)**	
Absence (≤ 44)	29 (39.2)
Presence (≥45)	45 (60.8)
**Dyadic sexual desire (SDI-2)**	
Mean *(SD)*	43.3 (16.5)
Medium/high (T2, T3)	45 (64.3)
Low (T1)^‡^	25 (35.7)
**Solitary sexual desire (SDI-2)**	
Mean *(SD)*	8.9 (8.3)
Medium/high (T2, T3)	44 (64.7)
Low (T1)^§^	24 (35.3)
**Skindex-17 symptoms**	
Not severe (0-49.9)	20 (27.8)
Severe (≥50.0)	52 (72.2)
**Skindex-17 psychosocial**	
Mild (≤ 20.82)	13 (18.1)
Moderate (20.83-37.5)	17 (23.6)
Severe (≥37.51)	42 (58.3)
**MCS-36**	
Mean *(SD)*	35.3 (10.0)
Median (IQR)	34.5 (27.7-43.2)
**PCS-36**	
Mean *(SD)*	40.9 (10.8)
Median (IQR)	43.3 (33.0-49.1)
**GHQ-12**	
0-3	38 (50.7)
≥4	37 (49.3)
**HADS-Anxiety**	
Normal	33 (46.5)
Borderline	10 (14.1)
Positive	28 (39.4)
**HADS-depression**	
Normal	46 (64.8)
Borderline	15 (21.1)
Positive	10 (14.1)

*SDQ, Sexual Dysfunction Questionnaire; SDI, Sexual Desire Inventory; SD, Standard Deviation; T, Tertile; MCS, Mental Component Summary; PCS, Physical Component Summary; GHQ, General Health Questionnaire; HADS, Hospital Anxiety and Depression Scale.*

† *Totals vary because of missing values.*

‡ *T1 defined as “low dyadic sexual desire (≤ 42)”.*

§ *T1 defined as “low solitary sexual desire (≤ 2)”*.

We performed different multivariate models (Table 3), each adjusted for sex and age plus variables associated with each of the four outcomes in univariate analyses (see Supplementary Tables 1a–c). In the first model, we found that high GHQ scores, and low BMI increased risk of sexual dysfunction, whereas family history of HS decreased risk of it. In the second model, high GHQ scores increased risk of low dyadic sexual desire. In third model, we found that low BMI, and poor mental status increased risk of low solitary sexual desire, whereas alcohol consumption decreased risk of it. In the final model, severe skin symptoms and high anxiety scores increased risk of hindered sexuality due to HS.

**Table 3:** Multivariate analysis for factors associated with sexual dysfunction, dyadic sexual desire, solitary sexual desire, hindered sexuality (logistic regression models).

	**Sexual dysfunction**			**Dyadic sexual desire**			**Solitary sexual desire**			**Hindered sexuality**	
	**OR (95%CI)^**†**^**	***P-*value**		**OR (95%CI)^**‡**^**	***P-*value**		**OR (95%CI)^**§**^**	***P-*value**		**OR (95%CI)^**¶**^**	***P-*value**
**Sex**											
Females	1			1			1			1	
Males	0.87 (0.24-3.11)	0.828		0.79 (0.24-2.65)	0.706		0.52 (0.11-2.53)	0.418		0.50 (0.17-1.48)	0.211
**Age**											
<25.0	1			1			1			1	
25.0-39.9	1.78 (0.44-7.21)	0.422		1.91 (0.45-8.05)	0.381		0.42 (0.07-2.51)	0.338		0.79 (0.20-3.17)	0.745
≥40.0	9.19 (1.38-61.2)	0.022		7.37 (1.46-37.2)	0.016		3.98 (0.56-28.2)	0.166		0.68 (0.15-3.10)	0.620
**BMI (kg/m2)**											
≤ 24.9	3.96 (1.11-14.1)	0.033		…			6.27 (1.30-30.2)	0.022		…	
≥25.0	1						1				
**Alcohol use**											
No							1				
Yes	…			…			0.06 (0.01-0.43)	0.004		…	
**Family history of HS**											
No	1										
Yes	0.04 (0.005-0.43)	0.007		…			…			…	
**GHQ-12**											
0-3	1			1							
≥4	7.04 (1.93-25.7)	0.003		5.02 (1.50-16.8)	0.009		…			…	
**MCS, tertiles**											
Medium/high (T2, T3)							1				
Low (T1)^††^	…			…			8.54 (1.65-44.0)	0.010		…	
**Skindex-17 symptoms**											
Not severe (0-49.9)										1	
Severe (≥50.0)	…			…			…			3.67 (1.13-12.0)	0.031
**HADS-Anxiety**											
Negative										1	
Positive	…			…			…			4.40 (1.25-15.5)	0.021

*HS, Hidradenitis Suppurativa; T, Tertile; OR, Odds Ratio; CI, Confidence Intervals; BMI, body mass index; GHQ, General Health Questionnaire; MCS, Mental Component Summary; HADS, Hospital Anxiety and Depression Scale.*

† *OR estimate the association of characteristics with presence of sexual dysfunction according to Sexual Dysfunction Questionnaire; ORs adjusted for sex, age, BMI, family history of HS and GHQ-12.*

‡ *OR estimate the association of characteristics with low dyadic sexual desire according to Sexual Desire Inventory-2; ORs adjusted for sex, age and GHQ-12.*

§ *OR estimate the association of characteristics with low solitary sexual desire according to Sexual Desire Inventory-2; ORs adjusted for sex, age, BMI, alcohol use and MCS tertiles.*

¶ *OR estimate the association of characteristics with hindered sexuality; ORs adjusted for sex, age, Skindex-17 symptoms and HADS-Anxiety.*

*T1 defined as “poor health status”*.

## Discussion

In this study, we found a high frequency of hindered sexuality and sexual dysfunction, and a high prevalence of medium/high levels of both dyadic and solitary sexual desire in patients with HS. Previous studies focused especially on sexual functioning impairment. For example, Alavi et al. (29) found that HS patients reported higher sexual dysfunction, impairment in sexual life, and higher sexual distress (i.e., concerns about sexuality), compared to healthy controls. This study also showed that sexual dysfunction predicted a decline in skin-related quality of life in HS patients when controlling for the effects of disease severity (29). Janse et al. (13) found that sexual health was impaired in HS patients, also showing that late onset of HS was associated with poor sexual function, whereas other clinical features, including anogenital involvement, early onset of HS, disease severity, and disease activity, were associated with worse QoL. The association between sexual functioning impairment and disease severity is still controversial. Our results did not show a higher risk of sexual dysfunction associated with increasing disease severity. These findings are consistent with previous studies (13, 14, 16, 29). For example, Kurek et al. (14) found that HS patients had higher sexual dysfunction and sexual distress compared to matched control subjects, whereas severity of cutaneous alterations correlated neither with sexual dysfunctions nor with sexual distress. Because of its clinical manifestations, HS is a debilitating and painful skin disease, which typically involves intimate body areas and affects patients' physical, social, and sexual health. We hypothesize that HS compromise patients' sexual life regardless of disease severity.

The presence of negative psychological functioning increased the risk of worse sexuality in our sample. Indeed, patients with minor psychiatric disorders had an increased risk of having sexual dysfunction and low dyadic sexual desire, whereas poor mental status increased the risk of low solitary sexual desire. Moreover, severe skin symptoms according to Skindex-17 and high anxiety were the only factors associated with an increased risk of hindered sexuality. Although previous literature reported associations between severe skin symptoms and both psychological distress (30) and quality of life (31), no previous studies examined the role these factors in negatively affecting sexuality in HS patients. Instead, Esmann and Jemec (7) suggested that severe skin symptoms may interfere with sexuality among HS patients. The authors found that some patients stopped their sexual life because of HS symptomatology and reported that their partners lost interest in them after HS lesions appearance. The latter study also found that HS patients were afraid of others' reaction (e.g., disgust) when they showed hidden parts of their body during sexual intercourse and they were embarrassed to explain their lesions.

Other factors decreased the risk of worse sexuality in our sample. For example, alcohol consumers among HS patients had a decreased risk of having low solitary sexual desire. Although previous research has examined the role of alcohol use in sexual behavior, little is known whether alcohol consumption is associated with sexual desire in dermatological patients. Commonly, alcohol use can influence individuals' sexual attitudes and behaviors, partly due to social disinhibition (32). Martin et al. (33) found that moderate alcohol use predicted a remission of low sexual desire among middle-aged and older men. Smith et al. (34) have shown that alcohol lowered inhibitions and increased the likelihood of engaging in sexual behaviors among women. We hypothesize that HS patients use alcohol effects to cope with severe physical symptoms (e.g., pain, itching, smell) and this, in turn, may increase solitary sexual desire. Moreover, patients with a family history of HS had a decreased risk of having sexual dysfunction compared to patients without a family history of HS. This result seems to indicate that having a family history of HS may provide social support, which can buffer against embarrassment, shame, and stigmatization that characterize HS patients' experiences (5), especially for sexual activities. This could indicate that family support for HS patients can help these individuals to cope with the burden of the disease. Surprisingly, we found that patients with low BMI had an increased risk of having sexual dysfunction and lower solitary sexual desire in comparison to patients with high BMI. In the general population the links between sexual function and BMI are unclear. Our results differ from those of previous studies, in which obese and overweight individuals reported an increased risk of sexual dysfunction (35, 36). In contrast, Kadioglu et al. (37) did not find significant differences between obese female and healthy control groups for sexual functioning and desire. Although little is known about these associations in HS patients, Theut Riis et al. (38) found that, in HS patients with low BMI, an increase in BMI was a predictor of increased patient-reported severity. Although higher BMI is associated with low sexual desire in the general population, according to Theut Riis et al. (38) we hypothesize that, in our study, HS patients with low BMI perceived worst physical health due to HS lesions appearance, and this, in turn, may have implications for sexual functioning and desire.

This study has some limitations. First, the small sample size limits the generalizability of these findings. However, HS is a rare disease and it is difficult to collect enough data to obtain statistically significant observations. Second, the cross-sectional design prevents us from making causal interpretations. Third, our findings rely exclusively on self-report measures, which raises the possibility of common method variance problems. The use of a multi-method approach (e.g., self-report and psychophysiological measures) is needed to confirm and extend our findings.

Notwithstanding these limitations, our study is among the first to examine the relationship among sexual desire and functioning, and general health status in patients with HS. Our results suggested that HS patients could have many problems in sexual function, while reporting good levels of sexual interest and desire. In this context, we found that many psychological factors, including minor psychiatric disorders, anxiety, and poor mental status, could increase the disease burden, predisposing to a greater risk of sexual difficulties. Our findings underline the important role of psychological aspects in HS patients, according to previous conclusions (3), and suggest that physicians should consider the impact of HS on patients' sexual health during assessment and management of the disease. A multidisciplinary therapeutic approach is recommended: in addition to medical prescriptions, HS patients could improve their mental and sexual functioning through specific psychological treatments, such as Cognitive-Behavioral Therapy and Educational Interventions, Sex Therapy, and Rational Emotive Therapy, which have been shown to be effective in previous studies (39). Given the importance of many psychological variables in HS sexual health, dermatologists should also pay more attention to different aspects of distress and suffering, in order to prevent a worsening in sexual function and mental health.

## Data Availability Statement

The raw data supporting the conclusions of this article will be made available by the authors, without undue reservation.

## Ethics Statement

The studies involving human participants were reviewed and approved by Comitato Etico IDI-IRCCS. The patients/participants provided their written informed consent to participate in this study.

## Author Contributions

RMQ and DA designed the study. RMQ, SM, FS, DA, and LI drafted the article. RMQ, LF, and RF administered the questionnaires and collected clinical data. SM and SF carried out the statistical analysis. All authors significantly contributed to the editing of manuscript, data interpretation, discussion, reviewed, and approved the final version for submission.

## Funding

This study was supported in part by the Progetto Ricerca Corrente 2019 of the Italian Ministry of Health, Rome, Italy.

## Conflict of Interest

The authors declare that the research was conducted in the absence of any commercial or financial relationships that could be construed as a potential conflict of interest.

## Publisher's Note

All claims expressed in this article are solely those of the authors and do not necessarily represent those of their affiliated organizations, or those of the publisher, the editors and the reviewers. Any product that may be evaluated in this article, or claim that may be made by its manufacturer, is not guaranteed or endorsed by the publisher.

## References

[1] Jemec GBE. Hidradenitis suppurativa. N Engl J Med. (2012) 366:158–64. 10.1056/NEJMcp1014163 22236226

[2] Machado MO Stergiopoulos V Maes M Kurdyak PA Lin P Wang L-J. Depression and anxiety in adults with hidradenitis suppurativa: a systematic review and meta-analysis. JAMA Dermatol. (2019) 155:939–45. 10.1001/jamadermatol.2019.0759 31166590 PMC6551580

[3] Misitzis A Goldust M Jafferany M Lotti T. Psychiatric comorbidities in patients with hidradenitis suppurativa. Dermatol Ther. (2020) 33:e13541. 10.1111/dth.13541 32385861

[4] Quinto RM Sampogna F Fania L Ciccone D Fusari R Mastroeni S. Alexithymia, psychological distress, and social impairment in patients with hidradenitis suppurativa. Dermatology. (2019) 19:1–8. 10.1159/000503319 31743903

[5] Sampogna F Fania L Mazzanti C Caggiati A Pallotta S Panebianco A. The broad-spectrum impact of hidradenitis suppurativa on quality of life: a comparison with psoriasis. Dermatology. (2019) 235:308–14. 10.1159/000496604 31121589

[6] Deckers IE Kimball AB. The handicap of Hidradenitis Suppurativa. Dermatol Clin. (2016) 34:17–22. 10.1016/j.det.2015.07.003 26617353

[7] Esmann S Jemec GBE. Psychosocial impact of hidradenitis suppurativa: a qualitative study. Acta Dermat Venereol. (2011) 91:328–32. 10.2340/00015555-1082 21394419

[8] Wilmoth MC. Sexuality: a critical component of quality of life in chronic disease. Nurs Clin North Am. (2007) 42:507-14. 10.1016/j.cnur.2007.08.008 17996752

[9] Arrington R Cofrancesco J Wu AW. Questionnaires to measure sexual quality of life. Qual Life Res. (2004) 13:1643–58. 10.1007/s11136-004-7625-z 15651536

[10] Ermertcan AT. Sexual dysfunction in dermatological diseases. J Eur Acad Dermatol Venereol. (2009) 23:999–1007. 10.1111/j.1468-3083.2009.03139.x 19250323

[11] Narang T Garima SMS. Psychosexual disorders and dermatologists. Indian Dermatol Online J. (2016) 7:149–58. 10.4103/2229-5178.182349 27294047 PMC4886584

[12] Sampogna F Abeni D Gieler U Tomas-Aragones L Lien L Titeca G. Impairment of sexual life in 3,485 dermatological outpatients from a multicentre study in 13 European countries. Acta Derm Venereol. (2017) 97:478–82. 10.2340/00015555-2561 27819713

[13] Janse IC Deckers IE van der Maten AD Evers AWM Boer J Van Der Zee HH. Sexual health and quality of life are impaired in hidradenitis suppurativa: a multicentre cross-sectional study. Br J Dermatol. (2017) 176:1042–7. 10.1111/bjd.14975 27534591

[14] Kurek A Peters EM Chanwangpong A Sabat R Sterry W Schneider-Burrus S. Profound disturbances of sexual health in patients with acne inversa. J Am Acad Dermatol. (2012) 67:422–8. 10.1016/j.jaad.2011.10.024 22182915

[15] Strada I Vegni E Lamiani G. Talking with patients about sex: results of an interprofessional simulation-based training for clinicians. Intern Emerg Med. (2016) 11:859–66. 10.1007/s11739-016-1468-9 27220953

[16] Cuenca-Barrales C Montero-Vílchez T Szepietowski JC Matusiak L Molina-Leyva A. Sexual impairment in patients with hidradenitis suppurativa: a systematic review. J Eur Acad Dermatol Venereol. (2020) 35:345–52. 10.1111/jdv.16726 32531099

[17] Levine SB. The nature of sexual desire: a clinician's perspective. Arch Sex Behav. (2003) 32:279–85. 10.1023/A:1023421819465 12807300

[18] American Psychiatric Association. Diagnostic and Statistical Manual of Mental Disorders: DSM-5 (5th ed.). Arlington: American Psychiatric Publishing, Inc (2013). p. 1092.

[19] Spector IP Carey MP Steinberg L. The sexual desire inventory: development, factor structure, and evidence of reliability. J Sex Marital Ther. (1996) 22:175–90. 10.1080/00926239608414655 8880651

[20] Hurley HJ. Axillary hyperhidrosis, apocrine bromhidrosis, hidradenitis suppurativa and familial benign pemphigus. Surgical approach. In: Roenigk RK, editor. Dermatologic Surgery. Principles and Practice. New York, NY: Marcel Dekker (1996). p. 623–45.

[21] Sartorius K Emtestam L Jemec GBE Lapins J. Objective scoring of hidradenitis suppurativa reflecting the role of tobacco smoking and obesity. Br J Dermatol. (2009) 161:831–9. 10.1111/j.1365-2133.2009.09198.x 19438453

[22] Zouboulis CC Tzellos T Kyrgidis A Jemec GBE Bechara FG Giamarellos-Bourboulis EJ. Development and validation of the International Hidradenitis Suppurativa Severity Score System (IHS4), a novel dynamic scoring system to assess HS severity. Br J Dermatol. (2017) 177:1401–9. 10.1111/bjd.15748 28636793

[23] Carlsson AM. Assessment of chronic pain. I. Aspects of the reliability and validity of the visual analogue scale. Pain. (1993) 16:87–101. 10.1016/0304-3959(83)90088-X 6602967

[24] Infrasca R. Sexual dysfunction questionnaire: scale development and psychometric validation. J Psychopathol. (2011) 17:253–60.

[25] Nijsten TE Sampogna F Chren MM Abeni D. Testing and reducing Skindex-29 using Rasch analysis: Skindex-17. J Invest Dermatol. (2006) 126:1244–50. 10.1038/sj.jid.5700212 16543899

[26] Piccinelli M Bisoffi G Bon MG Cunico L Tansella M. Validity and test- retest reliability of the Italian version of the 12-item General Health Questionnaire in general practice: a comparison between three scoring methods. Compr Psychiatry. (1993) 34:198–205. 10.1016/0010-440X(93)90048-9 8339539

[27] Iani L Lauriola M Costantini M. A confirmatory bifactor analysis of the hospital anxiety and depression scale in an Italian community sample. Health Qual Life Outcomes. (2014) 12:84–92. 10.1186/1477-7525-12-84 24902622 PMC4054905

[28] Apolone G Mosconi P. The Italian SF-36 Health Survey: translation, validation and norming. J Clin Epidemiol. (1998) 51:1025–36. 10.1016/S0895-4356(98)00094-8 9817120

[29] Alavi A Farzanfar D Rogalska T Lowes MA Chavoshi S. Quality of life and sexual health in patients with hidradenitis suppurativa. Int J Womens Dermatol. (2018) 4:74–9. 10.1016/j.ijwd.2017.10.007 30023423 PMC6047191

[30] Iani L Quinto RM Porcelli P Angeramo A-R Schiralli A Abeni D. Positive psychological factors are associated with better spiritual well-being and lower distress in individuals with skin diseases. Front Psychol. (2020) 11:552764. 10.3389/fpsyg.2020.552764 33123038 PMC7573544

[31] Abeni D Picardi A Pasquini P Melchi CF Chren MM. Further evidence of the validity and reliability of the Skindex-29: an Italian study on 2,242 dermatological outpatients. Dermatology. (2002) 204:43–9. 10.1159/000051809 11834849

[32] Dogan SJ Stockdale GD Widaman KF Conger RD. Developmental relations and patterns of change between alcohol use and number of sexual partners from adolescence through adulthood. Dev Psychol. (2010) 46:1747–59. 10.1037/a0019655 20677862 PMC2982773

[33] Martin SA Atlantis E Lange K Taylor AW O'Loughlin P Wittert GA. Predictors of sexual dysfunction incidence and remission in men. J Sex Med. (2014) 11:1136-47. 10.1111/jsm.12483 24548342

[34] Smith G Toadvine J Kennedy A. Women's perceptions of alcohol-related sexual disinhibition: personality and sexually-related alcohol expectancies. Int J Sex Health. (2009) 21:119–31. 10.1080/19317610902973241

[35] Esposito K Giugliano F Ciotola M De Sio M D'Armiento M Giugliano D. Obesity and sexual dysfunction, male and female. Int J Impot Res. (2008) 20:358–65. 10.1038/ijir.2008.9 18401349

[36] Silva BM Rêgo LM Galvão MA de Menezes Toledo Florêncio TM Cavalcante JC. Incidence of sexual dysfunction in patients with obesity and overweight. Rev Col Bras Cir. (2013) 40:196–202. 10.1590/S0100-69912013000300006 23912366

[37] Kadioglu P Yetkin DO Sanli O Yalin AS Onem K Kadioglu A. Obesity might not be a risk factor for female sexual dysfunction. BJU Int. (2010) 106:1357–61. 10.1111/j.1464-410X.2010.09348.x 20394615

[38] Theut Riis P Saunte DM Benhadou F. Low and high body mass index in hidradenitis suppurativa patients—different subtypes?. J Eur Acad Dermatol Venereol. (2018) 32:307–12. 10.1111/jdv.14599 28940801

[39] Frühauf S Gerger H Schmidt HM Munder T Barth J. Efficacy of psychological interventions for sexual dysfunction: a systematic review and meta-analysis. Arch Sex Behav. (2013) 42:915–33. 10.1007/s10508-012-0062-0 23559141

