# Supplementary material for: Sexuality in Persons With Hidradenitis Suppurativa: Factors Associated With Sexual Desire and Functioning Impairment
Source: Front Psychiatry. 2021 Oct 7;12:729104. doi: 10.3389/fpsyt.2021.729104 (PMC8528950; doi:10.3389/fpsyt.2021.729104)
Supplement: Supplementary file 1 [file Data_Sheet_1.docx]

| Table S1a: Association between socio-demographic characteristics of 77 patients with HS and sexual dysfunction, dyadic sexual desire, solitary sexual desire, hindered sexuality | | | | | | | | | | | | | | | | | | | | | | | | | |
| --- | --- | --- | --- | --- | --- | --- | --- | --- | --- | --- | --- | --- | --- | --- | --- | --- | --- | --- | --- | --- | --- | --- | --- | --- | --- |
|  | Sexual dysfunction (SDQ) | | | | |  | | Dyadic sexual desire | | | | |  | | Solitary sexual desire | | | | |  | | Hindered sexuality | | | |
|  | presence (N=45) |  | absence (N=29) |  |  | | T1§ (N=25) | |  | T2-T3 (N=45) |  |  | | T1¶ (N=24) | |  | T2-T3 (N=44) |  |  | | yes (N=47) | |  | no (N=29) |  |
|  | N^†^(%) |  | N^†^(%) | *P*value‡ |  | | N^†^(%) | |  | N^†^(%) | *P*value‡ |  | | N^†^(%) | |  | N^†^(%) | *P*value‡ |  | | N^†^(%) | |  | N^†^(%) | *P*value‡ |
| Sex |  |  |  |  |  | |  | |  |  |  |  | |  | |  |  |  |  | |  | |  |  |  |
| females | 31 (68.9) |  | 17 (58.6) |  |  | | 18 (72.0) | |  | 26 (57.8) |  |  | | 20 (83.3) | |  | 25 (56.8) |  |  | | 34 (72.3) | |  | 15 (51.7) |  |
| males | 14 (31.1) |  | 12 (41.4) | 0.456 |  | | 7 (28.0) | |  | 19 (42.2) | 0.305 |  | | 4 (16.7) | |  | 19 (43.1) | **0.034** |  | | 13 (27.7) | |  | 14 (48.3) | **0.087** |
| Age |  |  |  |  |  | |  | |  |  |  |  | |  | |  |  |  |  | |  | |  |  |  |
| <25.0 | 10 (22.2) |  | 8 (27.6) |  |  | | 4 (16.0) | |  | 14 (31.1) |  |  | | 6 (25.0) | |  | 12 (27.3) |  |  | | 12 (25.5) | |  | 6 (20.7) |  |
| 25.0-39.9 | 20 (44.4) |  | 16 (55.2) |  |  | | 10 (40.0) | |  | 221 (48.9) |  |  | | 7 (29.2) | |  | 22 (50.0) |  |  | | 21 (44.7) | |  | 15 (51.7) |  |
| ≥40.0 | 15 (33.3) |  | 5 (17.2) | 0.352 |  | | 11 (44.0) | |  | 9 (20.0) | **0.095** |  | | 11 (45.8) | |  | 12 (27.3) | 0.130 |  | | 14 (29.8) | |  | 8 (27.6) | 0.866 |
| Education level, years |  |  |  |  |  | |  | |  |  |  |  | |  | |  |  |  |  | |  | |  |  |  |
| low (≤8) | 14 (311) |  | 7 (24.1) |  |  | | 9 (36.0) | |  | 11 (24.4) |  |  | | 9 (37.5) | |  | 10 (22.7) |  |  | | 12 (25.5) | |  | 10 (34.5) |  |
| medium (9-13) | 21 (46.7) |  | 12 (41.4) |  |  | | 10 (40.0) | |  | 20 (44.4) |  |  | | 7 (29.2) | |  | 22 (50.0) |  |  | | 23 (48.9) | |  | 10 (34.5) |  |
| high (>13) | 10 (22.2) |  | 10 (34.5) | 0.515 |  | | 6 (24.0) | |  | 14 (31.1) | 0.660 |  | | 8 (33.3) | |  | 12 (27.3) | 0.224 |  | | 12 (25.5) | |  | 9 (31.0) | 0.455 |
| BMI (kg/m^2^) |  |  |  |  |  | |  | |  |  |  |  | |  | |  |  |  |  | |  | |  |  |  |
| ≤24.9 | 23 (51.1) |  | 8 (28.6) |  |  | | 13 (52.0) | |  | 16 (36.4) |  |  | | 14 (60.9) | |  | 15 (34.1) |  |  | | 22 (46.8) | |  | 10 (35.7) |  |
| ≥25.0 | 22 (48.9) |  | 20 (71.4) | **0.088** |  | | 12 (48.0) | |  | 28 (63.6) | 0.218 |  | | 9 (36.1) | |  | 29 (65.9) | **0.042** |  | | 25 (53.2) | |  | 18 (64.3) | 0.470 |
| Smoking status |  |  |  |  |  | |  | |  |  |  |  | |  | |  |  |  |  | |  | |  |  |  |
| never smokers | 10 (22.2) |  | 4 (13.8) |  |  | | 7 (28.0) | |  | 7 (15.6) |  |  | | 6 (25.0) | |  | 7 (15.9) |  |  | | 8 (17.0) | |  | 6 (20.7) |  |
| current smokers | 29 (64.4) |  | 22 (75.9) |  |  | | 15 (60.0) | |  | 33 (73.3) |  |  | | 15 (62.5) | |  | 32 (72.7) |  |  | | 33 (70.2) | |  | 20 (69.0) |  |
| ex smokers (at least 6m) | 6 (13.3) |  | 3 (10.3) | 0.664 |  | | 3 (12.0) | |  | 5 (11.1) | 0.435 |  | | 3 (12.5) | |  | 5 (11.4) | 0.630 |  | | 6 (12.8) | |  | 3 (10.3) | 0.935 |
| Alcohol use |  |  |  |  |  | |  | |  |  |  |  | |  | |  |  |  |  | |  | |  |  |  |
| no | 31 (68.9) |  | 15 (51.7) |  |  | | 20 (80.0) | |  | 24 (53.3) |  |  | | 21 (87.5) | |  | 22 (50.0) |  |  | | 30 (63.8) | |  | 18 (62.1) |  |
| yes | 14 (31.1) |  | 14 (48.3) | 0.150 |  | | 5 (20.0) | |  | 21 (46.7) | **0.039** |  | | 3 (12.5) | |  | 22 (50.0) | **0.003** |  | | 17 (36.2) | |  | 11 (37.9) | 1.00 |
| Physical activity |  |  |  |  |  | |  | |  |  |  |  | |  | |  |  |  |  | |  | |  |  |  |
| no | 33 (73.3) |  | 18 (62.1) |  |  | | 20 (80.0) | |  | 27 (60.0) |  |  | | 18 (75.0) | |  | 27 61.4) |  |  | | 34 (72.3) | |  | 19 (65.5) |  |
| yes | 12 (26.7) |  | 11 (37.9) | 0.318 |  | | 5 (20.0) | |  | 18 (40.0) | 0.114 |  | | 6 (25.0) | |  | 17 (38.6) | 0.295 |  | | 13 (27.7) | |  | 10 (34.5) | 0.610 |
| SDQ, Sexual Dysfunction Questionnaire; SDI, Sexual Desire Inventory; T, Tertile; OR, Odds Ratio; CI, Confidence Intervals; IHS4, International HS Severity Score System. | | | | | | | | | | | | | | | | | | | | | | | | | |
| †: Totals vary because of missing values; ‡: Fisher's exact test; §: T1 defined as “low dyadic sexual desire (≤42)"; ¶: T1 defined as “low solitary sexual desire (≤2)".  Pvalues <0.10 in bold | | | | | | | | | | | | | | | | | | | | | | | | | |

| Table S1b: Association between clinical characteristics of 77 patients with HS and sexual dysfunction, dyadic sexual desire, solitary sexual desire, hindered sexuality | | | | | | | | | | | | | | | | | | | | | | | |
| --- | --- | --- | --- | --- | --- | --- | --- | --- | --- | --- | --- | --- | --- | --- | --- | --- | --- | --- | --- | --- | --- | --- | --- |
|  | Sexual dysfunction (SDQ) | | | |  | | Dyadic sexual desire | | | | | |  | | Solitary sexual desire | | | |  | Hindered sexuality | | | |
|  | presence (N=45) |  | absence (N=29) |  | |  | | T1§ (N=25) |  | T2-T3 (N=45) |  |  | | T1¶ (N=24) | |  | T2-T3 (N=44) |  |  | yes (N=47) |  | no (N=29) |  |
|  | N^†^(%) |  | N^†^(%) | *P*value‡ | |  | | N^†^(%) |  | N^†^(%) | *P*value‡ |  | | N^†^(%) | |  | N^†^(%) | *P*value‡ |  | N^†^(%) |  | N^†^(%) | *P*value‡ |
| Age at onset |  |  |  |  | |  | |  |  |  |  |  | |  | |  |  |  |  |  |  |  |  |
| <18 | 18 (40.0) |  | 13 (44.8) |  | |  | | 7 (28.0) |  | 24 (53.3) |  |  | | 8 (33.3) | |  | 23 (52.3) |  |  | 20 (42.5) |  | 11 (37.5) |  |
| ≥18 | 27 (60.0) |  | 16 (55.2) | 0.820 | |  | | 18 (72.0) |  | 21 (46.7) | **0.048** |  | | 16 (66.7) | |  | 21 (47.7) | 0.203 |  | 27 (57.5) |  | 18 (62.1) | 0.811 |
| Family history of HS |  |  |  |  | |  | |  |  |  |  |  | |  | |  |  |  |  |  |  |  |  |
| no | 43 (95.6) |  | 22 (75.9) |  | |  | | 23 (92.0) |  | 38 (84.4) |  |  | | 22 (91.7) | |  | 37 (84.1) |  |  | 42 (89.4) |  | 25 (86.2) |  |
| yes | 2(4.4) |  | 7 (24.1) | **0.024** | |  | | 2 (8.0) |  | 7 (15.6) | 0.474 |  | | 2 (8.3) | |  | 7 (15.9) | 0.476 |  | 5 (10.6) |  | 4 (13.8) | 0.725 |
| Hurley stage |  |  |  |  | |  | |  |  |  |  |  | |  | |  |  |  |  |  |  |  |  |
| I | 17 (37.8) |  | 8 (28.6) |  | |  | | 9 (36.0) |  | 15 (34.1) |  |  | | 10 (41.7) | |  | 14 (32.6) |  |  | 15 (32.6) |  | 10 (34.5) |  |
| II | 25 (55.6) |  | 19 (67.9) |  | |  | | 15 (60.0) |  | 27 (61.4) |  |  | | 13 (54.2) | |  | 28 (65.1) |  |  | 27 (58.7) |  | 18 (62.1) |  |
| III | 3 (6.7) |  | 1 (3.6) | 0.558 | |  | | 1 (4.0) |  | 2 (4.5) | 1.00 |  | | 1 (4.2) | |  | 1 (2.3) | 0.573 |  | 4 (8.7) |  | 1 (3.4) | 0.804 |
| Sartorius score |  |  |  |  | |  | |  |  |  |  |  | |  | |  |  |  |  |  |  |  |  |
| 0-49 | 28 (62.2) |  | 16 (57.1) |  | |  | | 16 (64.0) |  | 24 (54.6) |  |  | | 13 (54.2) | |  | 26 (60.5) |  |  | 25 (54.3) |  | 19 (65.5) |  |
| 50-99 | 11 (24.4) |  | 7 (25.0) |  | |  | | 5 (20.0) |  | 13 (29.5) |  |  | | 7 (29.2) | |  | 11 (25.6) |  |  | 12 (26.1) |  | 7 (24.1) |  |
| ≥100 | 6 (13.3) |  | 5 (17.9) | 0.890 | |  | | 4 (16.0) |  | 7 (15.9) | 0.737 |  | | 4 (16.7) | |  | 6 (13.9) | 0.823 |  | 9 (19.6) |  | 3 (10.3) | 0.509 |
| IHS4 |  |  |  |  | |  | |  |  |  |  |  | |  | |  |  |  |  |  |  |  |  |
| mild (≤3) | 10 (22.2) |  | 4 (14.3) |  | |  | | 6 (24.0) |  | 8 (18.2) |  |  | | 5 (20.8) | |  | 8 (18.6) |  |  | 8 (17.4) |  | 6 (20.7) |  |
| moderate (4-10) | 13 (28.9) |  | 9 (32.1) |  | |  | | 8 (32.0) |  | 12 (27.3) |  |  | | 5 (20.8) | |  | 15 (34.9) |  |  | 11 (23.9) |  | 11 (37.9) |  |
| severe (≥11) | 22 (48.9) |  | 15 (53.6) | 0.771 | |  | | 11 (44.0) |  | 24 (54.5) | 0.674 |  | | 14 (58.3) | |  | 20 (46.5) | 0.469 |  | 27 (58.7) |  | 12 (41.4) | 0.305 |
| Disease duration, y |  |  |  |  | |  | |  |  |  |  |  | |  | |  |  |  |  |  |  |  |  |
| <5 | 9 (20.0) |  | 6 (20.7) |  | |  | | 6 (24.0) |  | 7 (15.6) |  |  | | 4 (16.7) | |  | 7 (15.9) |  |  | 8 (17.0) |  | 7 (24.1) |  |
| 5-14 | 25 (55.6) |  | 14 (48.3) |  | |  | | 12 (48.0) |  | 25 (55.6) |  |  | | 13 (54.2) | |  | 23 (52.3) |  |  | 27 (57.5) |  | 12 (41.4) |  |
| ≥15 | 11 (24.4) |  | 9 (24.4) | 0.857 | |  | | 7 (28.0) |  | 13 (28.9) | 0.709 |  | | 7 (29.2) | |  | 14 (31.8) | 1.00 |  | 12 (25.5) |  | 10 (34.5) | 0.384 |
| Number of locations involved | |  |  |  | |  | |  |  |  |  |  | |  | |  |  |  |  |  |  |  |  |
| ≤2 | 28 (62.2) |  | 14 (50.0) |  | |  | | 15 (60.0) |  | 24 (54.6) |  |  | | 13 (54.2) | |  | 24 (55.8) |  |  | 23 (50.0) |  | 19 (65.2) |  |
| 3-4 | 12 (26.7) |  | 10 (35.7) |  | |  | | 7 (28.0) |  | 13 (29.5) |  |  | | 8 (33.3) | |  | 13 (30.2) |  |  | 17 (37.0) |  | 6 (20.7) |  |
| ≥5 | 5 (11.1) |  | 4 (14.3) | 0.615 | |  | | 3 (12.0) |  | 7 (15.9) | 0.941 |  | | 3 (12.5) | |  | 6 (14.0) | 1.00 |  | 6 (13.0) |  | 4 (13.8) | 0.338 |
| Involvement of groin region | |  |  |  | |  | |  |  |  |  |  | |  | |  |  |  |  |  |  |  |  |
| yes | 37 (82.2) |  | 22 (78.6) |  | |  | | 5 (20.0) |  | 9 (20.4) |  |  | | 4 (16.7) | |  | 9 (20.9) |  |  | 6 (13.0) |  | 8 (27.6) |  |
| no | 8 (17.8) |  | 6 (21.4) | 0.764 | |  | | 20 (80.0) |  | 35 (79.6) | 1.00 |  | | 20 (83.3) | |  | 34 (79.1) | 0.757 |  | 40 (87.0) |  | 21 (72.4) | 0.137 |
| SDQ, Sexual Dysfunction Questionnaire; SDI, Sexual Desire Inventory; T, Tertile; OR, Odds Ratio; CI, Confidence Intervals; IHS4, International HS Severity Score System. | | | | | | | | | | | | | | | | | | | | | | |  |
| †: Totals vary because of missing values; ‡: Fisher's exact test; §: T1 defined as “low dyadic sexual desire (≤42)"; ¶: T1 defined as “low solitary sexual desire (≤2)".  Pvalues <0.10 in bold | | | | | | | | | | | | | | | | | | | | | | | |

| Table S1c: Association between patient-reported quality of life, health status, psychological distress, anxiety and depression of 77 patients with HS and sexual dysfunction, dyadic sexual desire, solitary sexual desire, hindered sexuality | | | | | | | | | | | | | | | | | | | | | |  |
| --- | --- | --- | --- | --- | --- | --- | --- | --- | --- | --- | --- | --- | --- | --- | --- | --- | --- | --- | --- | --- | --- | --- |
|  | Sexual dysfunction (SDQ) | | | |  | Dyadic sexual desire | | | |  | Solitary sexual desire | | | |  | | Hindered sexuality | | | | |  |
|  | presence (N=45) |  | absence (N=29) |  |  | T1§ (N=25) |  | T2-T3 (N=45) |  |  | T1¶ (N=24) |  | T2-T3 (N=44) |  | |  | | yes (N=47) |  | no (N=29) |  | |
|  | N^†^(%) |  | N^†^(%) | *P*value‡ |  | N^†^(%) |  | N^†^(%) | *P*value‡ |  | N^†^(%) |  | N^†^(%) | *P*value‡ | |  | | N^†^(%) |  | N^†^(%) | *P*value‡ | |
| Skindex-17 symptoms |  |  |  |  |  |  |  |  |  |  |  |  |  |  | |  | |  |  |  |  | |
| not severe (0-49.9) | 9 (21.9) |  | 10 (35.7) |  |  | 7 (30.4) |  | 13 (31.0) |  |  | 5 (21.7) |  | 13 (31.7) |  | |  | | 7 (16.3) |  | 13 (46.4) |  | |
| severe (≥50.0) | 32 (78.1) |  | 18 (64.3) | 0.275 |  | 16 (69.6) |  | 29 (69.0) | 1.00 |  | 18 (78.3) |  | 28 (68.3) | 0.564 | |  | | 36 (83.7) |  | 15 (53.6) | **0.008** | |
| Skindex-17 psychosocial |  |  |  |  |  |  |  |  |  |  |  |  |  |  | |  | |  |  |  |  | |
| mild (≤20.82) | 5 (12.2) |  | 8 (28.6) |  |  | 2 (8.7) |  | 11 (26.2) |  |  | 0 (-) |  | 13 (31.7) |  | |  | | 2 (4.6) |  | 11 (39.3) |  | |
| moderate (20.83-37.5) | 6 (14.6) |  | 10 (35.7) |  |  | 6 (26.1) |  | 10 (23.8) |  |  | 4 (17.4) |  | 10 (24.4) |  | |  | | 11 (25.6) |  | 6 (21.4) |  | |
| severe (≥37.51) | 30 (73.2) |  | 10 (35.7) | **0.008** |  | 15 (65.2) |  | 21 (50.0) | 0.221 |  | 19 (82.6) |  | 18 (43.9) | **0.001** | |  | | 30 (69.8) |  | 11 (39.3) | **0.001** | |
| MCS-36 |  |  |  |  |  |  |  |  |  |  |  |  |  |  | |  | |  |  |  |  | |
| medium/high (T2, T3) | 25 (61.0) |  | 20 (76.9) |  |  | 15 (68.2) |  | 27 (65.9) |  |  | 9 (40.9) |  | 30 (76.9) |  | |  | | 25 (58.1) |  | 21 (80.8) |  | |
| low (T1)^††^ | 16 (39.0) |  | 6 (23.1) | 0.196 |  | 7 (31.8) |  | 14 (31.1) | 1.00 |  | 13 (59.1) |  | 9 (23.1) | **0.007** | |  | | 18 (41.9) |  | 5 (19.2) | **0.068** | |
| PCS-36 |  |  |  |  |  |  |  |  |  |  |  |  |  |  | |  | |  |  |  |  | |
| medium/high (T2, T3) | 28 (68.3) |  | 18 (69.2) |  |  | 18 (81.8) |  | 26 (63.4) |  |  | 15 (68.2) |  | 28 (71.8) |  | |  | | 28 (65.1) |  | 18 (69.2) |  | |
| low (T1)^‡‡^ | 13 (31.7) |  | 8 (30.8) | 1.00 |  | 4 (18.2) |  | 15 (36.6) | 0.159 |  | 7 (31.8) |  | 11 (28.2) | 0.778 | |  | | 15 (34.9) |  | 8 (30.8) | 0.796 | |
| GHQ-12 |  |  |  |  |  |  |  |  |  |  |  |  |  |  | |  | |  |  |  |  | |
| 0-3 | 17 (38.6) |  | 20 (71.4) |  |  | 8 (32.0) |  | 28 (65.1) |  |  | 7 (30.4) |  | 27 (62.8) |  | |  | | 19 (42.2) |  | 19 (65.5) |  | |
| ≥4 | 27 (61.4) |  | 8 (28.6) | **0.008** |  | 17 (68.0) |  | 15 (34.9) | **0.012** |  | 16 (69.6) |  | 16 (37.2) | **0.019** | |  | | 26 (57.8) |  | 10 (34.5) | **0.060** | |
| HADS-Anxiety |  |  |  |  |  |  |  |  |  |  |  |  |  |  | |  | |  |  |  |  | |
| negative | 22 (53.7) |  | 19 (70.4) |  |  | 13 (52.0) |  | 29 (69.1) |  |  | 12 (50.0) |  | 27 (65.9) |  | |  | | 21 (47.7) |  | 21 (80.8) |  | |
| positive | 19 (46.3) |  | 8 (29.6) | 0.210 |  | 12 (48.0) |  | 13 (30.9) | 0.197 |  | 12 (50.0) |  | 14 (34.1) | 0.294 | |  | | 23 (52.3) |  | 5 (19.2) | **0.011** | |
| HADS-Depression |  |  |  |  |  |  |  |  |  |  |  |  |  |  | |  | |  |  |  |  | |
| negative | 34 (82.9) |  | 25 (92.6) |  |  | 19 (76.0) |  | 39 (92.9) |  |  | 18 (75.0) |  | 38 (92.7) |  | |  | | 35 (79.6) |  | 25 (96.2) |  | |
| positive | 7 (17.1) |  | 2 (7.4) | 0.300 |  | 6 (24.0) |  | 3 (7.1) | **0.069** |  | 6 (25.0) |  | 3 (7.3) | **0.066** | |  | | 9 (20.4) |  | 1 (3.8) | **0.079** | |
| SDQ, Sexual Dysfunction Questionnaire; SDI, Sexual Desire Inventory; T, Tertile; OR, Odds Ratio; CI, Confidence Intervals; IHS4, International HS Severity Score System. | | | | | | | | | | | | | | | | | | | | |  | |
| †: Totals vary because of missing values; ‡: Fisher's exact test; §: T1 defined as “low dyadic sexual desire (≤42)"; ¶: T1 defined as “low solitary sexual desire (≤2)".  Pvalues <0.10 in bold | | | | | | | | | | | | | | | | | | | | | |  |
